# Validating infrared thermography for non-invasive estimation of internal body temperature in hatchling Mojave desert tortoises

**DOI:** 10.1093/conphys/coag053

**Published:** 2026-07-31

**Authors:** Thomas A Radzio, Talisin T Hammond, Ronald R Swaisgood, Melissa J Merrick

**Affiliations:** San Diego Zoo Wildlife Alliance, Beckman Center for Conservation Research, 15600 San Pasqual Valley Road, Escondido, CA 92027, USA; San Diego Zoo Wildlife Alliance, Beckman Center for Conservation Research, 15600 San Pasqual Valley Road, Escondido, CA 92027, USA; San Diego Zoo Wildlife Alliance, Beckman Center for Conservation Research, 15600 San Pasqual Valley Road, Escondido, CA 92027, USA; San Diego Zoo Wildlife Alliance, Beckman Center for Conservation Research, 15600 San Pasqual Valley Road, Escondido, CA 92027, USA

**Keywords:** Chelonian, climate change resilience, ecophysiology, ectotherm*, Gopherus agassizii*, thermal ecology

## Abstract

Quantifying thermoregulatory parameters, such as preferred and realized body temperatures, is essential for predicting ectotherm responses to climate change, and accurate estimates of internal body temperature are central to these efforts. In reptiles, cloacal temperature serves as the primary field- and laboratory-standard proxy for internal body temperature. Infrared thermography (IRT) is increasingly used to estimate internal body temperatures of small ectotherms because it minimizes disturbance to animal behaviour, thereby improving data quality. Although IRT has advantages over traditional contact methods, it relies on correlations between external and internal body temperatures, which can vary across taxa. To evaluate IRT’s efficacy for estimating internal temperature of small turtles, we compared surface and cloacal temperatures of hatchling Mojave desert tortoises (*Gopherus agassizii*) under warming, elevated temperature and cooling conditions on two laboratory thermal gradients spanning moderate to extreme temperature ranges. Among five measured shell and body regions, shell temperatures correlated most strongly with cloacal temperatures. IRT produced accurate estimates during elevated temperature and cooling phases but was less accurate during warming from low initial body temperatures, particularly under steeper thermal gradients. We also evaluated IRT in two additional contexts: hatchling rearing enclosures and a behavioural experiment. Shell and cloacal temperatures aligned closely in rearing enclosures but exhibited larger discrepancies in the behavioural experiment. Our findings suggest that IRT is a promising tool for non-invasive estimation of cloacal temperature in hatchling desert tortoises (25–101 g). This tool is most useful under moderate thermal conditions when body temperatures are near thermal equilibrium but should be used with caution during periods of rapid temperature change when surface and internal temperatures may diverge. Once validated within a given study system, IRT can provide a practical alternative to traditional methods for monitoring body temperature while reducing disturbance and handling stress that may alter natural thermal behaviour.

## Graphical Abstract

**Graphical Abstract:**
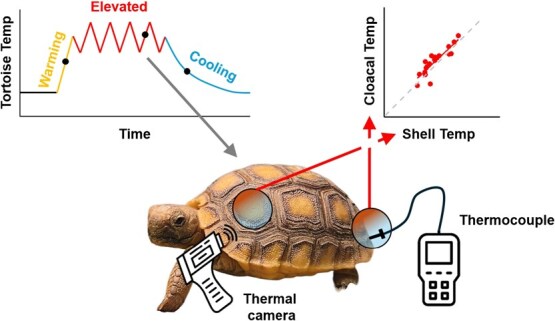

## Introduction

Understanding thermal requirements is critical for predicting habitat suitability and guiding conservation efforts of vulnerable species amid ongoing climate change (Angilletta 2009; Sinclair *et al.*, 2016). For ectotherms, key determinants of the thermal niche (temperatures that support positive population growth) are reflected by preferred body temperature range, realized field body temperatures and thermal sensitivity of performance (Gvoždík, 2018). Body temperature measurements are essential for quantifying these parameters, prompting researchers to develop and evaluate new methods that minimize observer effects on study animal behaviour while maintaining accuracy and improving the reliability of physiological inference (Tattersall, 2016).

Infrared thermography (IRT) has gained popularity as a non-invasive tool for measuring body temperatures, particularly in small ectotherms (Tattersall and Cadena, 2010; Tattersall, 2016). Compared to contact methods (e.g. cloacal thermocouples), IRT minimizes disturbance and enables temperature measurements of free-ranging individuals (Berg *et al.*, 2015). Other approaches, such as externally attached or surgically implanted temperature loggers, can provide continuous estimates of body temperature in larger individuals, but are not appropriate for very small tortoises, and externally attached loggers may only approximate body temperature once they have equilibrated with the animal. However, IRT measures surface rather than core temperatures, which are typically the physiological variable of interest. Thermal gradients throughout body tissues necessitate taxon-specific calibration studies to evaluate IRT’s reliability for internal temperature estimation before it can be confidently applied in conservation or ecological research contexts (Berg *et al.*, 2015; Barroso *et al.*, 2016; Thompson *et al.*, 2023; Spears *et al.*, 2024).

Although frequently used to study lizards, especially small-bodied species, IRT has rarely been applied to turtles, and we are not aware of work evaluating its performance for estimating internal body temperature in this group (Tattersall, 2016). The cylindrical body form of lizards limits differences between internal (i.e. cloacal) and skin temperatures in small individuals (Berg *et al.*, 2015; Barroso *et al.*, 2016; Spears *et al.*, 2024), whereas turtles’ more spherical morphology and insulating shell may amplify such discrepancies (McGinnis and Voigt, 1971). However, for small individuals, such as hatchlings, less pronounced body temperature gradients may make it possible to model relationships between internal and external temperatures.

IRT offers strong potential to advance studies of hatchling turtles, a critical life stage during which individuals are especially vulnerable to environmental stressors and whose thermal biology remains poorly understood. IRT is especially valuable for estimating parameters that require repeated, non-invasive observations of the same individual; these include preferred body temperature range and other physiobehavioural traits that can predict responses of young and vulnerable age classes to climate warming (Camacho and Rusch, 2017; Taylor *et al.*, 2021; Bodineau *et al.*, 2024). Beyond providing a non-invasive alternative for quantifying thermoregulatory parameters, IRT could also enable *in situ* studies of hatchling turtle thermal ecology, enhancing our understanding of their realized body temperatures, activity thresholds and performance in different thermal microhabitats relevant to habitat management and climate adaptation planning (Blais *et al.*, 2025).

Understanding thermal biology is especially important for predicting climate impacts on Mojave desert tortoise (*Gopherus agassizii*) populations, which are federally threatened and experiencing range-wide declines (Lovich *et al.*, 2014; Barrows *et al.*, 2016; Allison and McLuckie, 2018). Hatchlings may be particularly sensitive to climate change due to their high surface-area-to-volume ratios, rapid rates of water loss and limited capacity to buffer against environmental extremes relative to adults (Wilson *et al.*, 2001). Robust, minimally invasive methods for quantifying hatchling body temperature are therefore essential for linking thermal environments to performance and survival while minimizing stress to this early life stage.

Here, we compared surface thermographic and cloacal thermocouple measurements of hatchling Mojave desert tortoises across warming, elevated and cooling phases under two laboratory thermal gradient conditions. We further evaluated IRT’s performance across husbandry and behavioural experiment contexts, providing guidance on when this method most reliably approximates cloacal temperature. In these comparisons, we utilized cloacal temperature as a practical reference standard, acknowledging it as a proxy for, rather than a direct measurement of, true core temperature. Throughout the manuscript, cloacal temperature is therefore treated as a practical proxy for internal body temperature in hatchlings.

## Materials and methods

### Study animals

We studied 63 hatchling desert tortoises, collected as hatchlings or nearly fully developed eggs from nests laid in May–June 2022 by 15 wild females in a predator-proof enclosure at Edwards Air Force Base, located in the western Mojave Desert, CA. Eggs were incubated under controlled laboratory conditions until hatching. The hatchlings were reared in an indoor laboratory at The Living Desert Zoo and Gardens, closely following protocols outlined in Todd *et al.* (2021). Briefly, 50-gallon stock tanks (130 × 75 cm; Rubbermaid Home Products Inc., Atlanta, GA) fitted with sand/gravel substrate, plastic halfpipe burrows, a high-humidity hide, a 50-W daytime heat lamp, a 50-W nighttime ceramic heater and a UVB light maintained 5–6 hatchlings each. Heat sources and lights were positioned 30 cm above the substrate. Throughout this study, reported lamp heights refer to the distance from the bottom edge of the light fixture to the substrate. During the day, substrate temperatures typically ranged from 26 to 30°C in the cool end of enclosures and 37 to 40°C on the warm end. At night, enclosure temperatures decreased relative to daytime conditions. Food was provided 3 days per week, and water was provided 1 day per week.

All experiments were conducted between October 2022 and March 2023. Individuals were ~1–6 months post-hatching and spanned midline carapace lengths (MCL) of 46–78 mm and body masses of 25–101 g. Hatchlings were included in a study to examine individual recognition and therefore had temporary Sharpie identification marks on plastrons. However, for thermography trials, hatchlings were also marked with an Avery tag sticker placed on their second vertebral scute. Hatchlings were assigned to thermal gradient trials and laboratory application tests as described below. Trial-specific dates, sample sizes and size metrics are provided in Table S1. Study animals were fed, well-hydrated and growing rapidly.

### Temperature measurements and instrument accuracy

We measured hatchling surface temperatures using a FLIR E8-XT handheld thermal camera (Teledyne FLIR Inc., Goleta, CA) with the emissivity parameter set to 0.96 (Barroso *et al.*, 2016) and reflective temperature set to 35°C. We measured hatchling cloacal temperature using 40-gauge type K thermocouples and an Omega HH806AU high-accuracy (±0.05% of reading +0.3°C, NIST-traceable calibration) thermocouple reader (Omega Engineering Inc., Norwalk, CT). Thermocouples, thinly coated in epoxy, were lubricated with petroleum jelly and inserted 10–12 mm into the cloaca.

Although cloacal temperatures can differ greatly from deep core temperatures in large tortoises (>12 kg; McMaster and Downs, 2013), cloacal temperature is expected to more closely approximate internal thermal state in small-bodied individuals. Hatchlings in this study were much smaller, ranging from 25 to 101 g across experimental and laboratory application contexts. Cloacal temperature was used as a practical and widely accepted proxy for internal body temperature in small reptiles, rather than as a direct measure of true core temperature.

We validated thermal camera accuracy against the thermocouple reader by covering the thermocouple junction with two 1.5 × 1.5 cm pieces of black electrical tape (emissivity: 0.95) and comparing thermal camera and thermocouple measurements of tape temperature across a range (24–34°C) of slowly changing indoor air temperatures. Differences between thermal camera and thermocouple measurements were ≤0.5°C in 9 of 10 comparisons and 1.8°C in 1 comparison, consistent with manufacturer specifications (±2°C; Fig. S1).

### Thermal gradient trials

**Figure 1:**
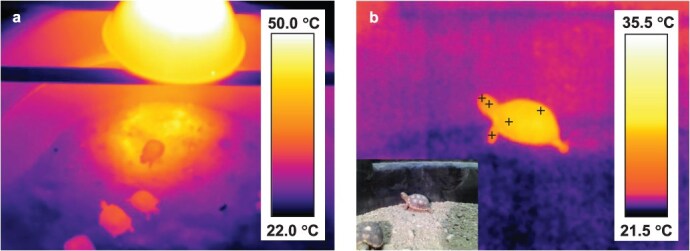
Representative thermographs from thermal gradient trials (expanded thermal gradient shown). (**a**) Warm end of the thermal gradient during the warming period; maximum substrate temperature directly beneath the lamp reached 43°C by 20 min after lamp onset. (**b**) Example thermograph illustrating the primary surface locations scored using the FLIR spot measurement tool for comparison with cloacal temperature: head (posterior to the eye), nose region, distal forelimb, first costal scute and third vertebral scute (crosshairs). Colour bars indicate the temperature range for each image.

Thermal gradient trials were conducted in hatchling home enclosures (described above), with hides, food dishes and UVB lights removed (Fig. 1). On the afternoon before each trial, one home enclosure (and its animals) was placed in a cool (~22°C) room without heat emitters so tortoises could acclimate overnight and begin trials the following morning at low body temperatures. Trials were conducted between 0900 and 1500 h, with five hatchlings from each enclosure tested simultaneously.

To evaluate IRT performance under warming, elevated and cooling temperature conditions while allowing behavioural thermoregulation, we conducted two sets of trials using a 150-W heat lamp: one elevated to 44 cm above the substrate (hereafter, the moderate thermal gradient; spanning ~22–45°C; *n* = 20 hatchlings) and another positioned 30 cm above the substrate (hereafter, the expanded thermal gradient; spanning ~22–60°C; *n* = 19 hatchlings; Fig. 1). Because gradients were generated by lamp onset and progressive substrate warming, most warming-phase measurements were collected at relatively low temperatures. For example, at 20 min into warming, maximum substrate temperatures directly beneath the lamp in the expanded-gradient trials were 40–45°C (Fig. 1).

The expanded thermal gradient was intentionally extended as a stringent validation scenario; upper gradient bounds of 60°C have been used in laboratory thermal gradient studies of reptiles (Tracy *et al.*, 2005; Sagonas *et al.*, 2017). This treatment also captured a broader range of warming trajectories early in trials, when the lamp heated rapidly but the substrate lagged, allowing us to sample a wider range of transient warming conditions while the gradient was still developing. Animals were continuously observed and could freely avoid extreme temperatures; exposure to the hottest substrate temperatures was voluntary and transient.

**Figure 2:**
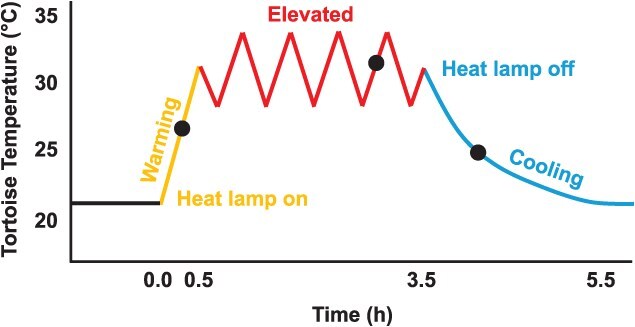
Schematic of temperature phases (warming, elevated and cooling) during thermal gradient trials. Heat lamp was turned on at time 0 to initiate warming and elevated temperature phases and turned off at ~3.5 h to initiate the cooling phase. Black dots represent example sampling times. In each phase, a thermograph was recorded, followed immediately by a cloacal temperature measurement. During the elevated phase, tortoise temperatures fluctuate to approximate a thermoregulating individual.

At the start of each trial, we recorded hatchling cloacal temperatures (range: 21.1–22.9°C). The heat lamp was turned on for ~3.5 h (warming and elevated phases) and then turned off (cooling phase; Fig. 2). We collected paired observations of hatchling surface and cloacal temperatures under warming, elevated and cooling temperature conditions (Fig. 2). For each paired measurement, we obtained a thermograph with the thermal camera, positioned ~40 cm from the animal at an ~45° oblique angle, immediately before (within ~30 s) measuring cloacal temperature with a thermocouple. The thermal camera saved a thermograph, and we later used the spot measurement tool in FLIR Research Studio software (Teledyne FLIR Inc., Goleta, CA) to score temperatures at five surface body locations: first costal scute, third vertebral scute, head (posterior to the eye), nose region and distal forelimb (Fig. 1). Nose region and forelimb temperatures were included to provide additional context on surface temperature variation across body regions. The right or left side of the animal was selected opportunistically, often depending on orientation of the individual relative to the camera and enclosure wall. Spot measurements were placed centrally within each region to avoid boundary pixels.

Warming and elevated temperature condition measurements were collected 5–83 min (median = 15) and 183–225 min (median = 195) after the heat lamp was turned on, respectively. Most (28 of 38) warming measurements occurred within 30 min of the heat lamp being turned on, but some were delayed considerably if tortoises initially spent a long time at the cool end of the gradient. Accordingly, warming observations reflect conditions during gradient development rather than at maximum gradient temperatures. Cooling temperature condition measurements were collected 5–140 min (median = 37) after the heat lamp was turned off (Fig. 2). Cooling measurements were collected across a wide range of times to ensure comprehensive sampling throughout cooling. A GoPro HERO 8 Black camera (GoPro Inc., San Mateo, CA) recorded all trials.

### Application tests

In addition to the thermal gradient trials, we compared IRT surface and thermocouple cloacal temperature measurements in two laboratory applications using older and larger hatchlings (Table S1): (i) rearing enclosures containing 5–6 hatchlings and (ii) a behavioural experiment containing two hatchlings per arena examining the effects of familiarity on hatchling interactions, burrow use and body temperatures. Because shell temperatures were the most reliable predictors of cloacal temperature, thermographs were collected from directly above each tortoise to approximate an overhead camera configuration commonly used for continuous behavioural monitoring.

The enclosures used for both rearing and the behavioural experiment contained sand/gravel substrate and were the same as those in the thermal gradient trials, but had 10-cm wide, half-pipe burrows and featured different heat sources. As described in the ‘Study Animals’ section, the rearing enclosures used a 50-W heat lamp suspended 30 cm above the substrate and three half-pipe burrows, producing substrate temperatures of 25–28°C at the cool end and 38–43°C at the warm end during observations. The behavioural experiment included two half-pipe burrows, each with only one open end. A 75-W heat lamp was suspended ~20 cm above the enclosure and angled at ~45° towards the entrance of one burrow. This configuration produced a steeper thermal gradient, with substrate temperatures of ~37–40°C immediately in front of the burrow entrance, ~24°C at the cool end of the burrow, and ~22°C in areas of the enclosure not directly exposed to the heat lamp.

### Data analysis

For thermal gradient experiment observations, we fit ordinary least-squares (OLS) linear regressions to evaluate relationships between surface (first costal scute, third vertebral scute, forelimb, head, and nose region) and cloacal temperatures during warming, elevated temperature and cooling phases, with each phase analysed separately. Because shell temperature (first costal and third vertebral scutes) explained the most variance in cloacal temperature, we focused on relationships between first costal scute and cloacal temperatures.

Elevated and cooling phases represent near steady-state thermal conditions in which shell and cloacal temperatures track closely. In addition to analysing these phases separately, data from elevated and cooling phases were also pooled to summarize predictive performance across the temperature states for which IRT is most applicable. Although each individual contributed only one observation per condition, pooling elevated and cooling phases introduced repeated measurements across conditions. To account for non-independence, pooled analyses were conducted using linear mixed-effects models with individual included as a random intercept, fit using restricted maximum likelihood. Because each individual contributed at most one observation per phase and between-individual variation in intercepts was minimal, random-intercept variance was estimated as zero in all pooled models (singular fits, as expected with the data structure). In cases where the estimated random-intercept variance was effectively zero (singular fit), the mixed-effects model was equivalent to an OLS regression; accordingly, we report standard OLS *r*^2^ from the corresponding simple linear regression for clarity and interpretability (Zuur *et al.*, 2009).

We also assessed regional heterogeneity under near steady-state conditions by comparing cloacal and surface temperatures during elevated temperature phases. Because each individual contributed simultaneous measurements across multiple body locations, we used linear mixed-effects models with body location as a fixed effect and individual included as a random intercept to account for repeated measures. When a significant effect of body location was detected, *post hoc* pairwise comparisons among locations were conducted using estimated marginal means with Tukey’s adjustment for multiple comparisons (*α* = 0.05).

To evaluate directional differences between surface and cloacal temperatures across thermal phases and gradient conditions, we calculated signed differences (first costal scute − cloacal temperature) and analysed these using a two-way linear model with thermal gradient, phase and their interaction as fixed effects. A mixed-effects model including individual as a random intercept was initially considered but resulted in singular fits (as expected); therefore, results from the equivalent OLS model are reported. Estimated marginal means were used to quantify mean differences within each gradient × phase combination, test whether differences deviated from zero, and compare gradients within phases and phases within gradients with appropriate multiple-comparison adjustments.

For application tests, we applied OLS regression to examine the relationship between first costal scute and cloacal temperatures.

Analyses were conducted using all available observations; records with missing values were excluded on a per-analysis basis when thermal image angle or tortoise posture (e.g. head withdrawn) prevented reliable scoring of head, nose region or forelimb temperatures (8% of thermal gradient observations). For all regressions, relationships were visually assessed using phase-specific scatterplots to evaluate linearity and the presence of influential observations. For first costal scute regressions and mixed-effects models, assumptions of linearity, homoscedasticity and approximate normality of residuals were evaluated using standard diagnostic plots and were generally met; formal normality tests (Shapiro–Wilk; Shapiro and Wilk, 1965) indicated mild departures from normality for the behavioural experiment regression, the moderate thermal gradient elevated + cooling phase regression and the moderate thermal gradient regional heterothermy model. However, residual Q–Q plots did not suggest severe deviations and inference was expected to be robust given the focus on mean differences and effect estimation. Analyses were conducted in R (v4.4.0; R Core Team, 2024) using RStudio (Posit Team, 2024), with linear mixed-effects models fit using the lme4 package (Bates *et al.*, 2015) and *P*-values and denominator degrees of freedom obtained using the lmerTest package, and *post hoc* comparisons conducted using the emmeans package (Lenth, 2024).

### Animal welfare

All procedures were approved by the San Diego Zoo Wildlife Alliance Institutional Animal Care and Use Committee (Protocol #21-002) and conducted under US Fish and Wildlife Service Federal Recovery Permit ES46010D-1.

## Results

In the thermal gradient trials, the first costal and third vertebral scutes were nearly equivalent as the strongest overall predictors of cloacal temperature during warming, elevated temperature and cooling phases under both gradient conditions, whereas relationships for non-shell surface temperatures (forelimb, head and nose region) were almost always weaker across phases and gradients (Table 1). We therefore focus on the predictive performance of the first costal scute, as analogous relationships for the third vertebral scute were very similar.

**Table 1:** Coefficients of determination (*r*^2^) from OLS regressions for relationships between thermocouple-measured cloacal temperatures and IRT surface temperatures during warming, elevated and cooling phases under moderate and expanded thermal gradient conditions

	**Warming**		**Elevated**		**Cooling**	
	**Moderate**	**Expanded**	**Moderate**	**Expanded**	**Moderate**	**Expanded**
First costal vs cloaca	**0.55**	0.19	**0.77**	**0.72**	**0.92**	**0.91**
Third vertebral vs cloaca	**0.50**	**0.25**	**0.73**	**0.65**	**0.91**	**0.90**
Forelimb vs cloaca	**0.29**	0.00	**0.36**	**0.42**	**0.60**	**0.77**
Head vs cloaca	**0.33**	0.26	**0.53**	**0.31**	**0.89**	**0.82**
Nose region vs cloaca	**0.39**	0.10	0.12	0.12	**0.72**	**0.79**

Bold values denote significant regressions (*P* < 0.05).

**Figure 3:**
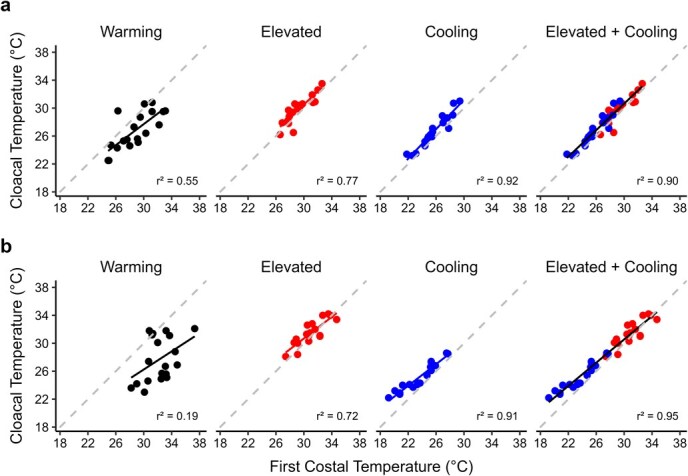
Relationships between first costal scute and cloacal temperatures during warming, elevated and cooling and elevated + cooling phases on (**a**) moderate (*n* = 20) and (**b**) expanded (*n* = 19) thermal gradients. The heat lamp was turned on for ~3.5 h (warming and elevated phases) and then turned off (cooling phase). Dashed lines indicate parity and solid lines indicate linear best fit. *r*^2^ values are from OLS regressions (see ‘Data Analysis’). All regressions, except for warming on the expanded gradient, were statistically significant (*P* < 0.05).

First costal scute temperature was a strong predictor of cloacal temperature during the combined elevated and cooling phases on both the moderate (*r*^2^ = 0.90, median absolute difference = 0.9°C, maximum absolute difference = 2.2°C, *n* = 40 total observations from 20 individuals; Fig. 3) and expanded (*r*^2^ = 0.95, median absolute difference = 1.1°C, maximum absolute difference = 3.1°C, *n* = 38 total observations from 19 individuals; Fig. 3) thermal gradients. However, during the warming phase, predictive performance was weaker, particularly on the hotter expanded thermal gradient (*r*^2^ = 0.19, median absolute difference = 5.2°C, maximum absolute difference = 8.2°C, *n* = 19 total observations, one per individual; Fig. 3).

**Figure 4:**
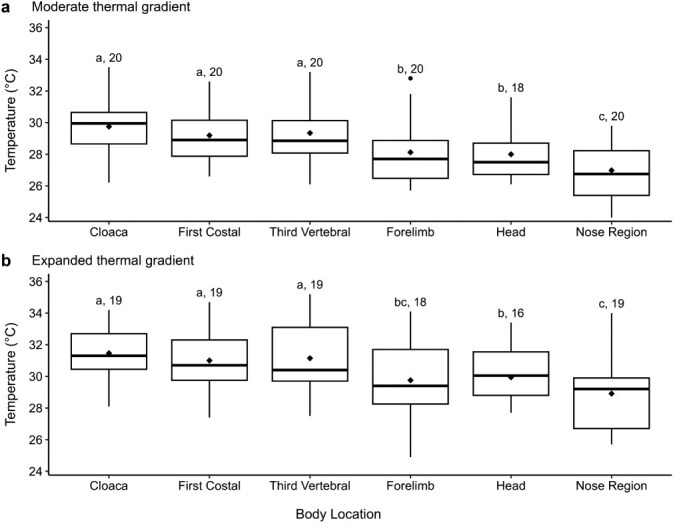
Boxplots of tortoise cloacal temperatures measured by thermocouple and body surface temperatures measured by IRT near the end of the elevated temperature phase under (**a**) moderate and (**b**) expanded thermal gradient conditions. Boxes show the median and interquartile range (IQR); whiskers extend to the most extreme values within 1.5 × IQR, and values beyond the whiskers are shown as outliers. Diamonds indicate estimated marginal means from linear mixed-effects models. Body locations sharing the same letter do not differ significantly (Tukey-adjusted pairwise comparisons); sample sizes for each body location are shown after the letter.

Regional heterothermy was observed during the elevated temperature phase under both thermal gradient conditions (moderate gradient: *F*_5,93.06_ = 30.09, *P* < 0.001; expanded gradient: *F*_5,86.03_ = 19.44, *P* < 0.001; Fig. 4). Head, nose region and forelimb temperatures were consistently lower than cloacal temperatures across both thermal gradient conditions, whereas shell and cloacal temperatures did not significantly differ (Tukey-adjusted pairwise comparisons; Fig. 4).

Across thermal phases, differences between first costal and cloacal temperatures varied in magnitude and direction (Fig. S2): first costal temperatures were higher during warming, were similar during the elevated phase, and were lower during cooling. Model-estimated mean differences were positive during warming (moderate gradient: +2.00°C; expanded gradient: +4.53°C), were small and did not differ from zero during elevated conditions (moderate: −0.56°C; expanded: −0.46°C), and were negative during cooling (moderate: −0.97°C; expanded: −1.54°C). These differences varied across phases and between thermal gradients (gradient × phase: *F*₂,₁₁₀ = 10.13, *P* < 0.001). The warming-phase offset was greater under the expanded gradient (mean difference = 2.53°C, *P* < 0.001), with no gradient differences during elevated or cooling phases (*P* ≥ 0.27).

**Figure 5:**
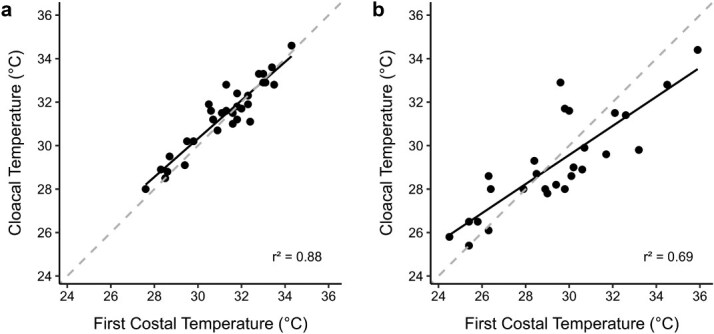
Relationships between first costal scute and cloacal temperatures in (**a**) rearing enclosures (*n* = 32) and in (**b**) the behavioural experiment (*n* = 27), evaluating the efficacy of IRT in two laboratory applications. Dashed lines represent parity. Solid lines represent linear best fit. Both regressions were statistically significant (*P* < 0.05).

In applied contexts, the rearing enclosure trial confirmed a strong relationship between first costal scute and cloacal temperatures (*r*^2^ = 0.88, median absolute difference = 0.4°C, maximum absolute difference = 1.5°C, *n* = 32; Fig. 5). The relationship was weaker in the behavioural experiment (*r*^2^ = 0.69, median absolute difference = 1.2°C, maximum absolute difference = 3.4°C, *n* = 27; Fig. 5). Tortoises in the behavioural experiment were older and larger than those in other analyses (Table S1; 25–101 g); however, body mass did not explain residual variation (linear regression: *P* = 0.52). Combining both application contexts yielded median and maximum absolute differences of 0.6 and 3.4°C, respectively. Notably, 55 of 59 observations across both applications fell within the thermal camera’s stated accuracy of ±2°C.

## Discussion

This study demonstrates that IRT can provide a reliable, non-invasive approximation of cloacal body temperatures in hatchling tortoises under elevated and cooling temperature conditions, provided discrepancies between shell and cloacal temperatures are acceptable for a given application. Shell surface recordings closely tracked cloacal temperatures during elevated and cooling phases across thermal gradient trials, husbandry enclosures and, though less strongly, within behavioural study arenas. Because it is consistently visible and well-defined, the shell is especially well suited for continuous or minimally disruptive thermographic video observations of individuals on thermal gradients. The ability of IRT to repeatedly and accurately estimate cloacal temperature in laboratory trials with minimal disturbance to study animals makes it a useful tool for a variety of applications in hatchling tortoises, including assessing thermal preference and other thermoregulatory parameters required to predict climate change impacts.

Although IRT performed well under elevated and cooling conditions, shell and cloacal temperatures showed greater divergence during warming, especially on the steeper expanded thermal gradient, where temperatures exceeded the typical range encountered by hatchlings in nature. This pattern was consistent with regional comparisons, in which all body regions were warmer than cloacal temperature during warming but generally cooler during elevated and cooling phases (Fig. S2). These results demonstrate that peripheral body regions can deviate systematically from core body temperature and respond more rapidly to changing environmental conditions than cloacal temperature consistent with documented regional heterothermy in reptiles (Roark and Dorcas, 2000; Du *et al.*, 2010). Our experimental design likely represented an especially challenging warming scenario for IRT because heating was driven primarily from above (heat lamp), which can rapidly elevate shell surface temperatures before internal tissues equilibrate. Such divergence is likely due to internal body temperature gradients that develop during rapid warming, a phenomenon potentially accentuated by the spherical body form and shell structure of turtles (McGinnis and Voigt, 1971). Discrepancies during warming may also have been amplified by basking behaviour early in trials, which likely accelerated surface heating relative to internal tissues. This result aligns with Berg *et al.* (2015) who also reported the greatest discrepancies between lizard skin and cloacal temperatures during periods of rapid warming.

While previous work by Barroso *et al.* (2016) in lizards found that eye temperatures closely tracked cloacal temperatures during warming, head surface temperatures in hatchling tortoises did not provide a comparably strong proxy for cloacal temperature, and the eye was not consistently distinguishable as a thermally distinct feature in our thermographs. Future validation work could test whether alternative regions improve inference during rapid warming. In particular, the shoulder region may provide a useful compromise. It is often accessible to thermography but may warm more similarly to internal tissues than the shell itself.

IRT-based inference of internal temperature may be best suited for small ectotherms, in which body temperature gradients are minimal (Stevenson, 1985). Mojave desert tortoise hatchlings used in thermal gradient trials were ~1–2.5 months old and had already grown substantially to 29–54 g. It seems likely that smaller hatchlings, either of this species or others that inherently produce smaller young (e.g. snapping turtles: <10 g, Congdon *et al.,* 1999), would show stronger correlations between external and cloacal body temperatures during rapid warming (Stevenson, 1985). Although IRT-based body temperature estimation performs best on dry surfaces, these findings remain relevant to hatchlings of aquatic and marine Chelonian species, which typically begin life on land. More broadly, these results should not be assumed to generalize across turtle species without taxon- and size-specific validation.

The capacity of IRT to continuously monitor body temperature without disturbing study animals holds great promise for turtle thermal ecology research. Whereas traditional invasive probes can alter hatchling behaviour, IRT minimizes observer effects and offers a less intrusive option for studies requiring repeated measurements of undisturbed individuals (Bodineau *et al.*, 2024). Another valuable advantage of this method is that thermographic data can be captured as images or videos, allowing for greater flexibility during postprocessing. Building on this, researchers have developed automated pipelines for extracting animal temperature data from IRT videos (Afonso *et al.*, 2024). Potential climate-related applications include investigating how incubation temperature and physiological state influence hatchling thermal preference, performance and critical early-life events such as nest dispersal and burrow excavation (Congdon *et al.*, 1999; Blumberg *et al.*, 2002). Field deployment of IRT may be problematic for burrow-dwelling species, for which direct or continuous observation may be challenging; in such cases, miniature data loggers or temperature-sensitive transmitters affixed to the shell may be more practical (Berg *et al.*, 2015).

Overall, our results show that IRT can provide a useful approximation of cloacal temperature in hatchling Mojave desert tortoises, particularly for smaller individuals evaluated in controlled thermal-gradient trials (≤61 mm MCL; ≤54 g), during elevated and cooling conditions where surface and cloacal temperatures remained closely aligned. However, rapid warming, especially under steep gradients, drove shell and cloacal temperatures apart. In these cases, the method was not as reliable. Although the thermal camera has a stated accuracy of ±2°C, calibration against a certified thermocouple reader indicated closer agreement under our experimental conditions (Fig. S1). Even so, this level of uncertainty may still be consequential for applications requiring precise estimates of body temperature or narrow thermal thresholds. The trade-off between accuracy and disturbance should guide the application of IRT. For studies where repeated, non-invasive temperature readings are prioritized over precise cloacal measurements, IRT offers clear advantages, especially in controlled environments. Future work should test this approach in smaller hatchlings and across species and directly compare IRT- and thermocouple-derived thermoregulatory parameter estimates to quantify the impact of measurement error on ecological models.

## Supplementary Material

Web_Material_coag053

## Data Availability

Data and R code are available in a Figshare repository: https://doi.org/10.6084/m9.figshare.31271974.
