# Supplementary material for: Validating infrared thermography for non-invasive estimation of internal body temperature in hatchling Mojave desert tortoises
Source: Conserv Physiol. 2026 Jul 31;14(1):coag053. doi: 10.1093/conphys/coag053 (PMC13427760; doi:10.1093/conphys/coag053)
Supplement: Web_Material_coag053 [file web_material_coag053.zip › Radzio_et_al_supplemental_materials_clean.docx]

**Supplemental Materials**

Table S1. Trial-specific test dates, sample sizes (n), and hatchling size metrics (mass and midline carapace length; mean, SD, minimum, and maximum) for desert tortoises used across experiments. Hatchlings used in the moderate and expanded thermal gradient trials were non-overlapping individuals.

**Mass (g)**

| **Trial** | **Dates** | **n** | **Mean** | **SD** | **Min** | **Max** |
| --- | --- | --- | --- | --- | --- | --- |
| **Expanded thermal gradient trials** | Oct 11, 2022–Oct 14, 2022 | 19 | 35 | 3.9 | 29 | 44 |
| **Moderate thermal gradient trials** | Nov 9, 2022–Nov 18, 2022 | 20 | 41 | 7.1 | 29 | 54 |
| **Rearing enclosures** | Jan 4, 2023–Jan 17, 2023 | 32 | 56 | 11.6 | 27 | 79 |
| **Behavioral experiment** | Jan 5, 2023–Mar 2, 2023 | 27 | 64 | 18.2 | 25 | 101 |

**Midline carapace length (mm)**

| **Trial** | **Dates** | **n** | **Mean** | **SD** | **Min** | **Max** |
| --- | --- | --- | --- | --- | --- | --- |
| **Expanded thermal gradient trials** | Oct 11, 2022–Oct 14, 2022 | 19 | 51 | 2.1 | 48 | 56 |
| **Moderate thermal gradient trials** | Nov 9, 2022–Nov 18, 2022 | 20 | 55 | 3.7 | 49 | 61 |
| **Rearing enclosures** | Jan 4, 2023–Jan 17, 2023 | 32 | 62 | 5.1 | 47 | 72 |
| **Behavioral experiment** | Jan 5, 2023–Mar 2, 2023 | 27 | 65 | 7.7 | 46 | 78 |


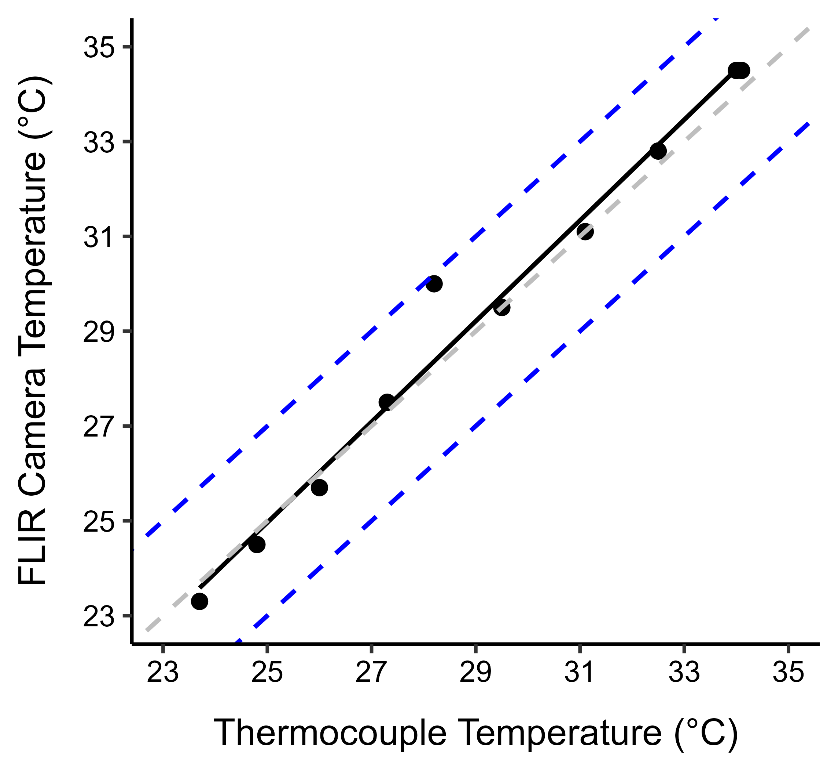


Figure S1. Temperatures of black electrical tape taken with the certified thermocouple reader and the FLIR thermal camera used in this study demonstrated good agreement between the two devices (n = 10). Solid black line represents ordinary least-squares regression fit. Grey dashes represent one-to-one line, and blue dashes represent the thermal camera’s manufacturer stated accuracy of ±2 °C with respect to the one-to-one line.


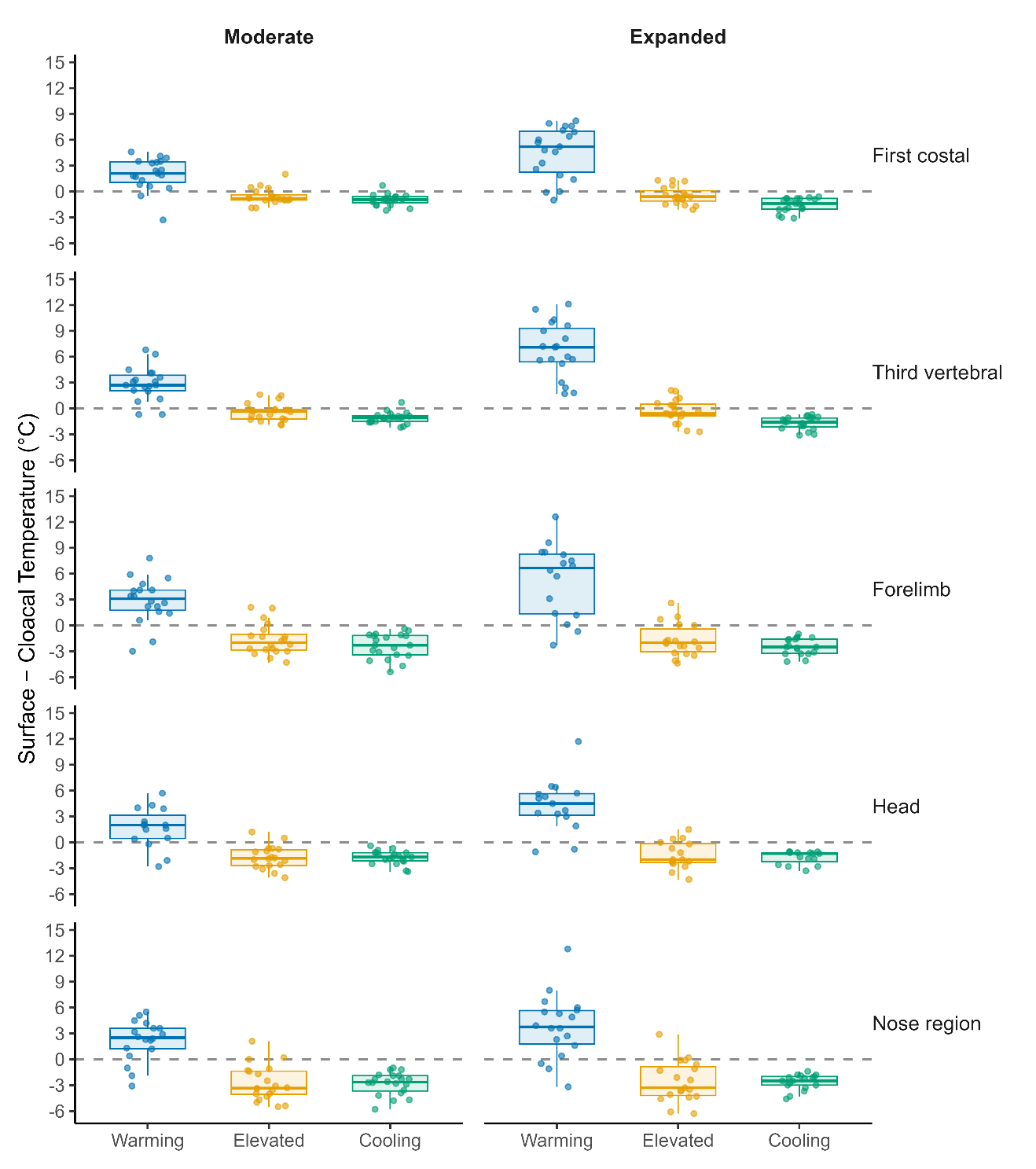


Figure S2. Differences between surface and cloacal temperatures across thermal phases and gradients. Boxplots show the distribution of temperature offsets (surface − cloacal, °C) for five anatomical regions (first costal, third vertebral, forelimb, head, and nose region) under moderate (left) and expanded (right) thermal gradients. Points represent individual observations; horizontal lines indicate medians and interquartile ranges (IQR), and whiskers extend to 1.5 × IQR. The dashed line denotes zero difference (no offset between surface and cloacal temperature). Across regions, surface temperatures were higher than cloacal temperatures during warming and lower during cooling; during the elevated phase, surface temperatures were approximately equal to cloacal temperatures for the first costal and third vertebral scutes but lower for other regions. The magnitude of positive offsets during warming was greater under the expanded gradient, whereas differences between gradients were not detected during the elevated and cooling phases.
