# Supplementary figures and images for: Validating infrared thermography for non-invasive estimation of internal body temperature in hatchling Mojave desert tortoises
Source: Conserv Physiol. 2026 Jul 31;14(1):coag053. doi: 10.1093/conphys/coag053 (PMC13427760; doi:10.1093/conphys/coag053)

**Graphical Abstract**

**
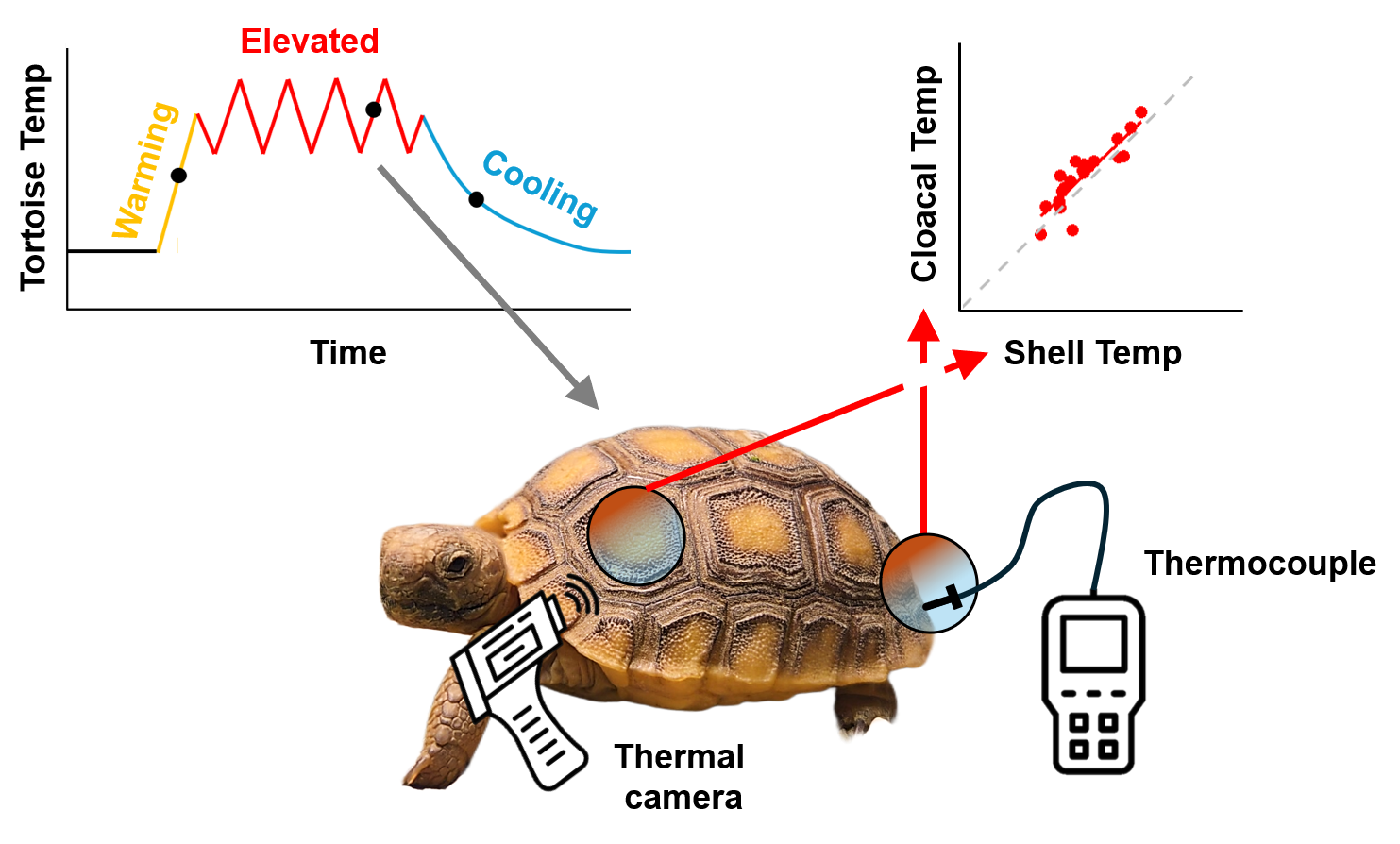
**

Supplement: Web_Material_coag053 [file web_material_coag053.zip › Radzio et al graphical abstract.docx]
